# Highly Connected Populations and Temporal Stability in Allelic Frequencies of a Harvested Crab from the Southern Pacific Coast

**DOI:** 10.1371/journal.pone.0166029

**Published:** 2016-11-04

**Authors:** Noemi Rojas-Hernandez, David Veliz, Marcela P Riveros, Juan P. Fuentes, Luis M. Pardo

**Affiliations:** 1 Departamento de Ciencias Ecológicas, Instituto de Ecología y Biodiversidad (IEB), Núcleo Milenio en Ecología y Manejo Sustentable (ESMOI), Facultad de Ciencias, Universidad de Chile, Santiago, Chile; 2 Instituto de Ciencias Marinas y Limnológicas, Facultad de Ciencias, Universidad Austral de Chile, Valdivia, Chile; 3 Centro de Investigación de Dinámica de Ecosistemas Marinos de Altas Latitudes (IDEAL), Valdivia, Chile; National Cheng Kung University, TAIWAN

## Abstract

For marine invertebrates with a benthic adult form and a planktonic larva phase, the connectivity among populations is mainly based on larval dispersal. While an extended larval phase will promote gene flow, other factors such as an intensive fishery and geographical barriers could lead to changes in genetic variability. In this study, the population genetic structure of the commercial crab *Metacarcinus edwardsii* was analyzed along 700 km of the Chilean coast. The analysis, based on eight microsatellite loci genotyped from megalopae and adult crabs, considered temporal and spatial patterns of genetic variation. The results showed no evidence of spatial patterns in genetic structure, suggesting high connectivity among the sampling sites. The temporal analysis showed no evidence of changes in allele frequencies and no evidence of a recent bottleneck. The lack of spatial structure and allele variation over time could be explained by the interaction of factors such as i) low reproductive variance due to the capability of females to store sperm in the seminal receptacle, which can be used for successive broods, ii) high larval dispersal and iii) high individual reproductive output. Using our data as priors, a genetic modelling approach coincided, predicting this temporal and spatial stability. The same analysis showed that a reduction in population size leads to the loss of genetic variability in populations, as well as of the genetic cohesiveness between populations, pointing out the importance management for species under exploitation, such as *M*. *edwardsii*.

## Introduction

Most benthic marine invertebrates are sessile or have little movement in their adult stage; dispersion happens during the planktonic larval period [1,2]. Typically, in this process a level of larval self-recruitment occurs in populations of origin, as well as an exchange of individuals among geographically separate populations [3]. The behavior of the larvae favors horizontal migration along the coast and vertical migration, promoting larval retention in nearshore waters [4,5,6].

Population structure cannot be predicted only by the mode of larval development and the extent of the planktonic period [7]. Species without a planktonic larval phase usually show higher levels of population genetic differentiation than species with planktonic larval development. However, species with planktonic larvae may have small-scale spatial structure, for example the starfish *Patiriella regularis*, where upwelling acts as a barrier to gene flow [8]. On the other hand, there are species with direct development that have genetic homogeneity over significant spatial scales, such as the gastropods *Littorina sitkana* and *Nucella lapillus*, where larval transport is aided by rafting [7, 9]. In this context, larval dispersal involves interactions between larval behavior and ocean transport [10]. Therefore, the genetic structure of marine populations is not only influenced by the mode of development, but also by oceanographic conditions such as tides, coastal upwelling [11,12], winds, physical barriers and life history traits [13].

Species with long lived pelagic larvae do not always show homogenous genetic patterns in time and space. For example, temporal variations in allele frequencies have been described [14,15] and related to a phenomenon called chaotic patchiness [16,17]. Several hypotheses try to explain the factors that can cause genetic heterogeneity, for example strong genetic drift caused by high mortality during early development, the action of natural selection during the larval stage and catastrophic events in the marine environment could change the pattern of larval recruitment in different years [18]. The possibility of low larvae mixture [19] and high reproductive variance [20] may also occur.

For species facing commercial fishing pressure, population size is an important factor influencing genetic variability and population connectivity [21]. Genetic variability is related to population size in relation to the effect of the genetic drift [22]. It would be expected that populations with reduced size will have lower genetic variability and changes in allele frequencies generation by generation. Changes in allele frequencies and allele diversity have been well described in marine species under exploitation [23,24], however other species have shown temporal stability in genetic diversity despite fishing pressure [25,26]. Therefore, it is important to determine the significance of effective population size in genetic stability and population cohesiveness.

Another important point to consider for the genetic structure of populations is the presence of biogeographic barriers and the phylogeographic breaks. Well-known phylogeographic breaks in the Pacific are located at Point Conception (34.5° N) in California [27], 30–32° S in northern [28] and 42° S in southern Chile [29]. While studies performed in Chile have identified species limited by the break at 30–32° S [30,31], other species did not align with these biogeographic breaks [32,31].

While these studies focused on phylogeographic analysis with a historical component, for harvested species the actual patterns of connectivity must be understood. In this context, decapod crustaceans are a good model to study connectivity due to their high fertility and extended larval development. In this study we performed a population genetic analysis on the crab *Metacarcinus edwardsii*, a crustacean inhabiting from the coast of Guayaquil, Ecuador to the Beagle Canal in Chile [33]. In Chile, *M*. *edwardsii* represents around 70% of national crab landings in artisanal fisheries [34]; exploitation is concentrated mainly around Chiloe Island in southern Chile [35]. The pelagic larval development of this species lasts three months (at 14±0.5°C) with five zoea larval stages and one megalopa stage [36] that settle mainly between November and December [37]. Adults inhabit subtidal environments, feeding on carrion and bivalves [38].

While previous studies have described aspects of the species reproductive biology [39,40], larva identification [41] and the effects of the fisheries on the reproductive potential of males [35], this information must be related to population genetics studies in order to be integrated in measures of protection for exploited populations. Our study covered 700 km of coastline, considering different sites and individuals sampled over 4 different years, in order to describe the spatial and temporal genetic structure of *M*. *edwardsii*.

## Materials and Methods

### Sampling, DNA extraction and microsatellite amplification

**Fig 1:**
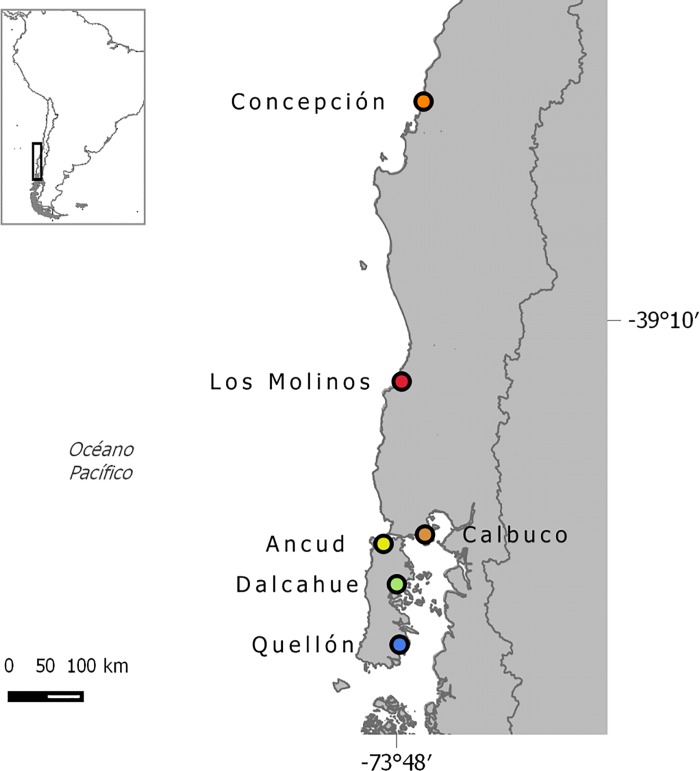
*Metacarcinus edwardsii* sampling sites.

To study the spatial genetic structure, 291 adult crabs were sampled from six localities: Concepción (CO), Los Molinos (LM), Ancud (AN), Calbuco (CA), Dalcahue (DA) and Quellón (QU) (Fig 1). Samples were obtained by Scuba diving during 2011 to 2014. To study the temporal genetic variability a total of 157 megalopa larvae were collected from Los Molinos (LM) during four years (2009 to 2012) using a passive larva collector, described by Pardo et al. 2010 [42]. Each megalopa was identified using the description of Pardo et al. (2009b) [41] and stored in 95% ethanol until analysis. Sample size and year of sampling are shown in Table 1. All collections and analyses were conducted in Chile and complied with its existing laws (Resolución Exenta No. 3088 Subsecretaría de Pesca).

**Table 1:** Sample size by site and year.

Group	Locality	Coordinates	Year	N	Code
Adult	Los Molinos (LM)	39°51′16.7″ S; 73°23′40.3″O	2011	24	LM 2011
			2013	24	LM 2013
	Dalcahue (DA)	42°22′46.3″ S; 73°35′42.5″O	2011	24	DA 2011
			2013	24	DA 2013
	Ancud (AN)	41°50′59.8″ S; 73°51′32.5″O	2012	37	AN 2012
			2013	24	AN 2013
	Calbuco (CA)	41°45′47.1″ S; 73°05′20.1″O	2012	16	CA 2012
			2013	16	CA 2013
	Quellón (QU)	43°08′18.4″ S; 73°36′43.4″O	2012	47	QU 2012
			2013	29	QU 2013
	Concepción (CO)	36°21'6.75"S; 72°50'52.68"O	2014	26	CO 2014
Megalopae	Los Molinos (LM)	39°51′16.7″ S; 73°23′40.3″O	2009	35	Megalopae 2009
			2010	51	Megalopae 2010
			2011	37	Megalopae 2011
			2012	34	Megalopae 2012

Genomic DNA was extracted from adult crabs using the salt method described by Aljanabi and Martinez (1997) [43]. DNA was extracted from megalopae using the QIAamp DNA Mini Kit (QIAGEN) and purified with sodium acetate (3M, pH 5.2). Eight polymorphic microsatellite loci were amplified following the procedure of Rojas-Hernández et al. (2014) [44]. Fragment analysis was performed using an Applied Biosystems 3100 sequencer at the Pontificia Universidad Católica de Chile. The allelic data matrix was built using the Peak Scanner software (Applied Biosystems).

### Adult data analysis

The number of alleles per locus, expected (*H*_E_) and observed (*H*_O_) heterozygosity were estimated with the GENETIX software [45]. With this same software, 5000 permutations on monolocus genotypes were used to test linkage disequilibrium and 5000 allele permutations were used to test for departures from Hardy Weinberg Equilibrium (HWE). FSTAT software [46] was used to estimate the allelic richness, while MICROCHECKER software [47] was used to identify possible null alleles in the microsatellite loci data.

To ensure that the analysis was performed with unrelated individuals, the r_xy_ index [48] was estimated with IDENTIX software [49]. IDENTIX used multilocus data to estimate the relatedness between pairs of individuals within each locality, compared with a distribution of simulated genotypes constructed after an allele permutation among individuals. 1000 permutations were generated to build this simulated distribution.

When highly variable genetic markers are used for the study of population structure, an alternative to the F_ST_ index is recommended [50]. One such index is G”_ST_; which is not influenced by the heterozygosity of samples. Thus, population structure was evaluated using four different methods: i) F_ST_ with GENETIX software, ii) G”_ST_ with GenAlEx software [51], iii) a Bayesian approximation with STRUCTURE software [52] and iv) an iterative reassignment of individuals with FLOCK software [53]. The statistical significance of the global F_ST_ was evaluated with GENETIX software using 5000 permutations. The GenAlEx software allowed the estimation of the global G”_ST_ and its significance was evaluated with 9999 permutations. The STRUCTURE software infers the most probable number of populations; the analysis was performed using the admixture model and the correlated allele frequencies model. The procedure was run 5 times for each K estimation (from K = 1 to K = 11) with a burn-in of 200000 MCMC iterations. LnP(D) values obtained for each K value were compared using the Structure Harvester software [54]. FLOCK software randomly divides the collection of genotypes into K genetic groups; the genotypes are reassigned to the group with the highest probability of belonging at each iteration following the multilocus method of maximum likelihood described by Paetkau et al. (1995) [55]. For our analysis, 50 runs and 30 iterations were used for values of K = 2 to 6 with a LLOD value ranging from 0 to 0.6.

To assess association between genetic differentiation and geographic distance, a Mantel test was performed using the GENETIX software. The statistical significance was tested with 5000 permutations. Since temporal comparisons within each locality showed no significant difference, data from each locality were pooled for posterior spatial analysis. In order to determine the migration patterns of *M*. *edwardsii* among localities, historical migration rates were estimated using the MIGRATE software [56]. MIGRATE uses a coalescent approach to estimate mutation-scaled migration rates (M) for each population over the last 4Ne generations. The analysis used maximum likelihood, the Brownian motion mutation model and the matrix migration model containing 10 short chains of 40000 steps and three long chains of 400000 steps, after a burn-in step of 40000 and a static heating scheme of 6 chains with increasing temperatures (1, 1.25, 1.67, 2.5, 5 and 1e^6^; the swapping interval was 1). Mutation rates of 1 x 10^−2^ and 1 x 10^−3^ were used to estimate the migration rate.

#### Effective population size

The effective population size was estimated using two different methods. First, the Bayesian method was implemented in ONeSAMP software [57] with the default settings. The second was the LD method implemented in NeEstimator [58]. In addition, a Kruskal-Wallis test was performed in order to compare Nb and Ne values obtained from both software.

#### Searching for evidence of a recent bottleneck

BOTTLENECK software [59] was used to detect evidence of a recent genetic bottleneck. Briefly, BOTTLENECK estimates the likelihood of recent reductions in effective population size by comparing the expected heterozygosity under HWE with the expected heterozygosity under mutation-drift equilibrium. The two phase mutation model with a 70% stepwise mutation was used.

### Megalopa data analysis

Four cohorts of megalopae were sampled in Los Molinos from 2009 to 2012 (Table 1). The number of alleles per locus, linkage disequilibrium, H_E,_ H_O_ and departures from HWE were estimated for each year with the GENETIX software. To determine differences among the cohorts analyzed, F_ST_ and G''_ST_ were estimated in the same way as for adults. Two analyses were performed: the first comparing the four megalopa cohorts and the second including each megalopa cohort and the adults collected in the same locality. STRUCTURE and FLOCK software were also used to detect differences among megalopa cohorts with same settings as in the adult analysis.

Taking into account that larval retention near the coast and the reproductive variance could generate non-random larval settlement in the same area, the likelihood of detecting related individuals in a sample was estimated using the r_xy_ value and the statistical significance was obtained with 1000 permutations in IDENTIX software.

#### Effective population size

For iteroparous species, estimates of effective population size need to consider samples composed of individuals of different ages. Therefore, the estimated population size of each cohort is interpreted as an approximation of the actual number of breeders per season (Nb), instead of an effective population size per generation (Ne) [60]. Considering this difference, Nb was estimated for each megalopa cohort and an approximate Ne was estimated using pooled data from these four cohorts. These calculations were done with ONeSAMP and NeEstimator software.

### Simulation of the effect of population size and the migration rate

For economically important species, exploring the effects of change in both population size and migration rate is relevant in order to understand population connectivity and changes in genetic variability. Considering that no spatial structure was observed in *M*. *edwardsii* and there was no evidence for a recent bottleneck, we explored the factors that would affect this pattern in a scenario of increased fishing. Currently, around five thousand tons of this species are harvested every year from southern Chile, affecting the population size [35]. Migration rates could be influenced by changes in both oceanographic circulation and upwelling processes [61]. Thus, this simulation tested for an effect of a reduction in population sizes and migration rates among sampling sites. The simulated datasets were built using EASYPOP 2.0.1 software [62] considering the following conditions: diploid organism, two sexes, random mating system, 6 sampling sites, equal number females and males in each generation, island migration model, eight loci with free recombination, a 0.01 microsatellite mutation rate, a mixed mutation model with Kam = 0.3, allelic states = 24 (similar to the mean allele number observed in this study) and maximum variability of the initial population. Three variables were tested simultaneously in the simulation: i) population size: 100000, 50000, 10000, 5000, 1000 and 500 individuals, ii) migration rate: 10% (as observed in this study), 1%, 0.1%, 0.01% and 0.001%, and iii) three different time horizons: after 10, 50 and 100 generations.

## Results

### Analysis of adults

All loci were polymorphic; the mean allele richness varied from 6.29 (Cedw15) to 25.44 (Cedw4) with a mean of 24 alleles per locus (S1 Table). Departures from HWE were detected in three out of eight microsatellite loci (α = 0.01), but deviations were not associated with any specific locus, sampling period or location. Null alleles do not appear to be responsible for the observed departures from HWE. Ten out of the 168 comparisons showed evidence for linkage disequilibrium (α = 0.01), however this disequilibrium was not observed in the same pair of alleles at all sites. The mean r_xy_ values estimated for the groups of individuals collected in the same year showed no statistical evidence (Permutation, P > 0.05) for highly related individuals. This evidence suggests that samples are composed of unrelated individuals (S1 Table).

For the temporal genetic structure, F_ST_ and G”_ST_ estimated for samples obtained in the same locality but in different years showed no significant differences: LM (F_ST_ = 0.001, P = 0.366 and G”_ST_ = 0.026, P = 0.325), DA (F_ST_ = 0.005, P = 0.078 and G”_ST_ = 0.067, P = 0.067), AN (F_ST_ = -0.003, P = 0.933 and G”_ST_ = -0.035, P = 0.924), CA (F_ST_ = -0.008, P = 0.949 and G”_ST_ = -0.082, P = 0.942) and QU (F_ST_ = 0.005, P = 0.068 and G”_ST_ = 0.002, P = 0.523). Considering these results, the next analyses were performed with pooled data from each locality.

**Fig 2:**
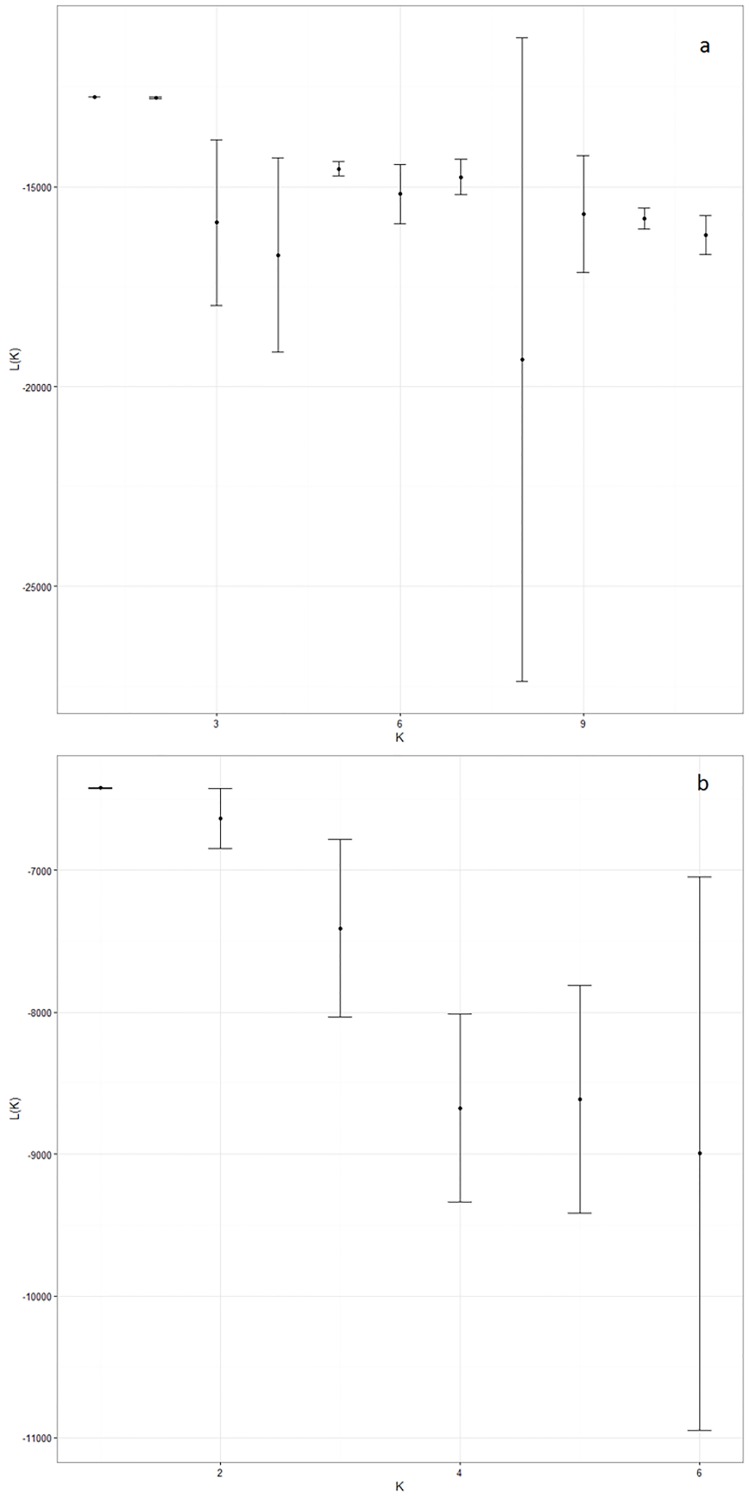
Inference of the number of the genetic clusters estimated through the L(K). (a.) adults collected from six localities. (b.) megalopae collected at Los Molinos. Each K represents the mean and standard deviation of five independent runs.

For the spatial genetic structure, the global F_ST_ and G”_ST_ showed no significant differences among sampling sites (F_ST_ = 0.001, P = 0.147 y G”_ST_ = 0.006, P = 0.293). The Structure Harvester showed K = 1 had the highest likelihood L(K) mean value and the lowest standard deviation (Fig 2A). FLOCK software showed the same pattern; the individual assignments in two or more reference groups showed no statistical significance for all runs performed with different LLOD values. It is important to note that FLOCK does not include K = 1 in the analysis. Duchesne and Turgeon (2012) [52] indicate that an indecisive result must be interpreted as an absence of population structure or a low power of the analysis due to the low number of loci or alleles. Considering the high number of alleles found and reasonable sample sizes used in this study, the analysis suggests no spatial or temporal structure in *M*. *edwardsii*. While, with regards to the effect of distance, the Mantel test showed no evidence of an association between genetic distance and geographic distance (Mantel test, Z = 33.86, P = 0.1496).

#### Effective population size and recent bottleneck

The Ne estimated were similar between sites, ranging from 16 (LM 2013) and 475 (DA 2011), except for QU 2013 that presented an extreme value of 1574 (Table 2). A Kruskal-Wallis test comparing Nb with Ne values from two software programs showed no significant differences (p = 0.338). Evidence for a recent bottleneck was found in one (DA 2011, P = 0.006) out of 11 possible estimations (CO 2014, P = 0.320, LM 2011, P = 0.371, LM 2013, P = 0.156, DA 2013, P = 0.273, AN 2012, P = 0.098, AN 2013, P = 0.473, QU 2012, P = 0.098, QU 2013, P = 0.156, CA 2012, P = 0.098, CA 2013, P = 0.125).

**Table 2:** Nb and Ne estimated with ONeSAMP and NeEstimator for adults and megalopae collected from the different sampling sites and years.

			OneSamp		NeEstimator	
		n	Ne	Confidence interval	Ne	Confidence interval
Adult	DA 2011	24	475.17	264.83–1185.00	196.7	112.2–708.5
	DA 2013	19	205.45	109.81–512.36	49.4	35.4–79.2
	LM 2011	18	132.37	76.73–280.68	78.2	56.4–124.2
	LM 2013	23	16.68	12.88–22.55	44.8	33.5–65.9
	AN 2012	35	38.23	30.30–49.25	323	190.5–987.3
	AN 2013	22	20.43	15.64–27.94	155.2	94.7–400.8
	QU 2012	32	196.77	133.64–334.86	359.9	205.5–1312.8
	QU 2013	25	1574.59	643.27–5932.69	177.9	110.7–427.4
	CA 2012	16	232.31	122.54–672.29	25.1	20.4–32.1
	CA 2013	14	190.96	92.85–531.93	49.2	33.7–87.2
	CO 2014	24	148.35	89.59–289.64	40.1	34–48.4
Megalopa larvae	Megalopae 2009	25	61.28	42.39–92.39	∞	226.8 - ∞
	Megalopae 2010	38	153.30	95.09–256.62	571.2	194.7 - ∞
	Megalopae 2011	23	315.32	155.67–909.01	1345	122.1 - ∞
	Megalopae 2012	29	10.85	9.05–13.04	118.2	65.5–488.3
	All (≈Ne)	111	198.58	167.62–239.01	∞	∞

#### Migration between pairs of localities

The mutation-scaled historical migration rates (*M*) ranged from 1.1 to 3.2 (Table 3) and the estimates of Θ ranged from 0.2 to 7.4 (LM = 6.1255, DA = 5.4971, AN = 6.0260, CA = 4.2633, QU = 4.4672, CO = 6.5019). There was no tendency related to a reduction in migration rate for geographically distant pairs of localities. Considering the mutation rate of 1 x 10^−2^, the migration rate between localities ranged from 0.01 to 0.031, with a total immigration rate of 0.1 for each location. While, when the mutation rate was 1 x 10^−3^, the migration rate between localities ranged from 0.001 to 0.003, with a total immigration rate of 0.01 for each location. Considering this result, the mean for self-recruitment (estimated as 1- total immigration rate) ranged from 0.90 (with a mutation rate of 1 x 10^−2^) to 0.99 (with a mutation rate of 1 x 10^−3^).

**Table 3:** Number of migrants per generation (with 95% CI) of *Metacarcinus edwardsii* by sampling site.

From/to	LM	DA	AN	CA	QU	CO
LM		2.964 (3.175–3.590)	1.350 (1.490–1.638)	2.541 (2.736–3.083)	1.512 (1.659–1.815)	1.528 (1.671–1.823)
DA	3.246 (3.040–3.461)		2.549 (2.740–2.941)	1.091 (1.180–1.316)	2.457 (2.645–3.022)	1.434 (1.573–1.721)
AN	1.066 (0.882–1.315)	3.036 (3.249–3.472)		1.867 (2.034–2.381)	1.421 (1.564–1.716)	2.016 (2.180–2.353)
CA	2.920 (2.723–3.125)	1.276 (1.415–1.564)	1.970 (2.138–2.315)		2.279 (2.460–2.650)	1.373 (1.508–1.738)
QU	1.339 (1.208–1.479)	2.753 (2.956–3.169)	1.242 (1.377–1.630)	2.657 (2.8567–3.215)		1.666 (1.815–1.973)
CO	1.8186 (1.665–1.981)	1.200 (1.335–1.635)	2.412 (2.598–2.794)	1.704 (1.864–2.243)	1.580 (1.822–2.065)	

### Analysis of megalopa larvae

Seven out of eight microsatellites were used for the megalopa analysis. The locus Cedw4 showed low quality and was excluded for all analyses performed for the megalopa. The seven loci used for megalopae showed high polymorphism with values ranging from 4.92 (Cedw15) to 12.57 (Cedcrab3) (S2 Table). While 1 out of 82 comparisons between pairs of loci showed evidence for linkage disequilibrium (α = 0.01), this finding was not consistent across sampling years.

In the temporal analysis performed for the four years of samples obtained at Los Molinos, the global analysis showed no evidence for differences in allele frequencies (F_ST_ = 0.0017, P = 0.1536 and G”_ST_ = 0.016, P = 0.180). Even when the analysis was performed with the adults collected at the same location, no temporal structure was detected (F_ST_ = 0.0016, P = 0.1218 and G”_ST_ = 0.011, P = 0.241). This result is consistent with that obtained with STRUCTURE (Fig 2B) and FLOCK software; both suggested K = 1 as the most probable number of genetic groups. Furthermore, there was no evidence of related individuals for any of the four cohorts analyzed (S2 Table), suggesting that zoea larvae belonging to the same litter are separated early in water column.

#### Effective population size

The effective number of breeders (Nb) was calculated for each cohort (megalopae 2009 to 2012) and the effective population size (Ne) was estimated pooling all megalopa data. Nb and Ne had a similar range of variation and even some values of Nb were higher than values of Ne estimated for the adults sampled in the same locality (Los Molinos) (Table 2).

### Modelling of changes in population size and reciprocal migration

The simulation started with 6 sites containing 100,000 individuals and an average of 24 alleles per locus. The estimated *Ne* in generation t = 1 for each of these six sampling sites had an average Ne = 476, similar to the Ne values obtained with our data.

**Fig 3:**
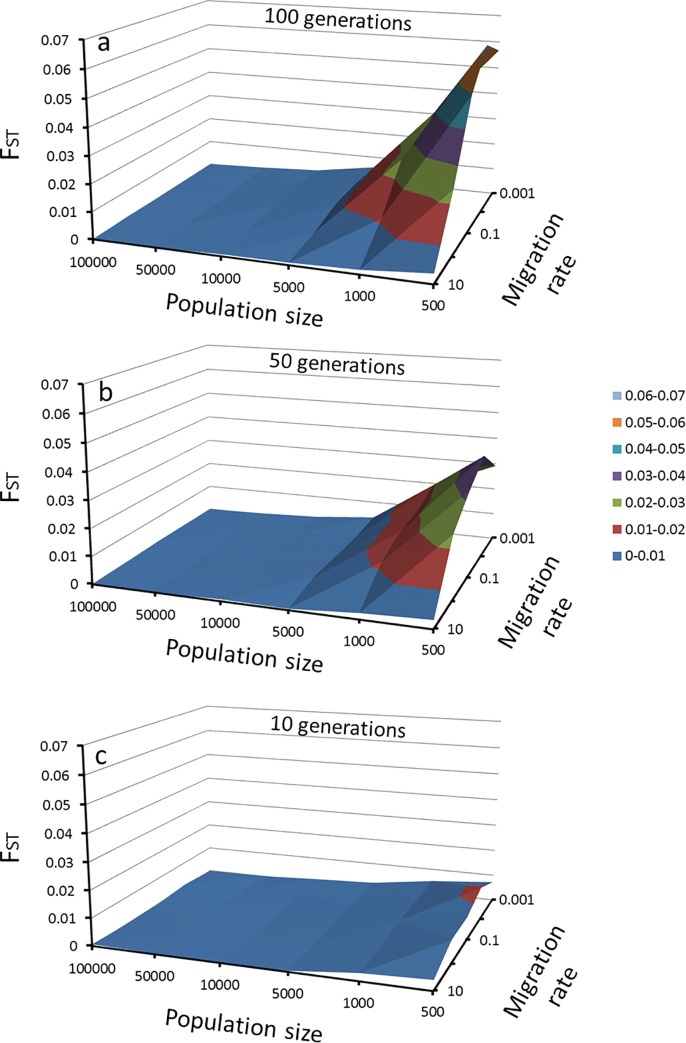
**Changes in F_ST_ estimated for the simulation of six populations as a function of the population size and migration rate, data after a) 100, b) 50 and c) 10 generations.**

**Fig 4:**
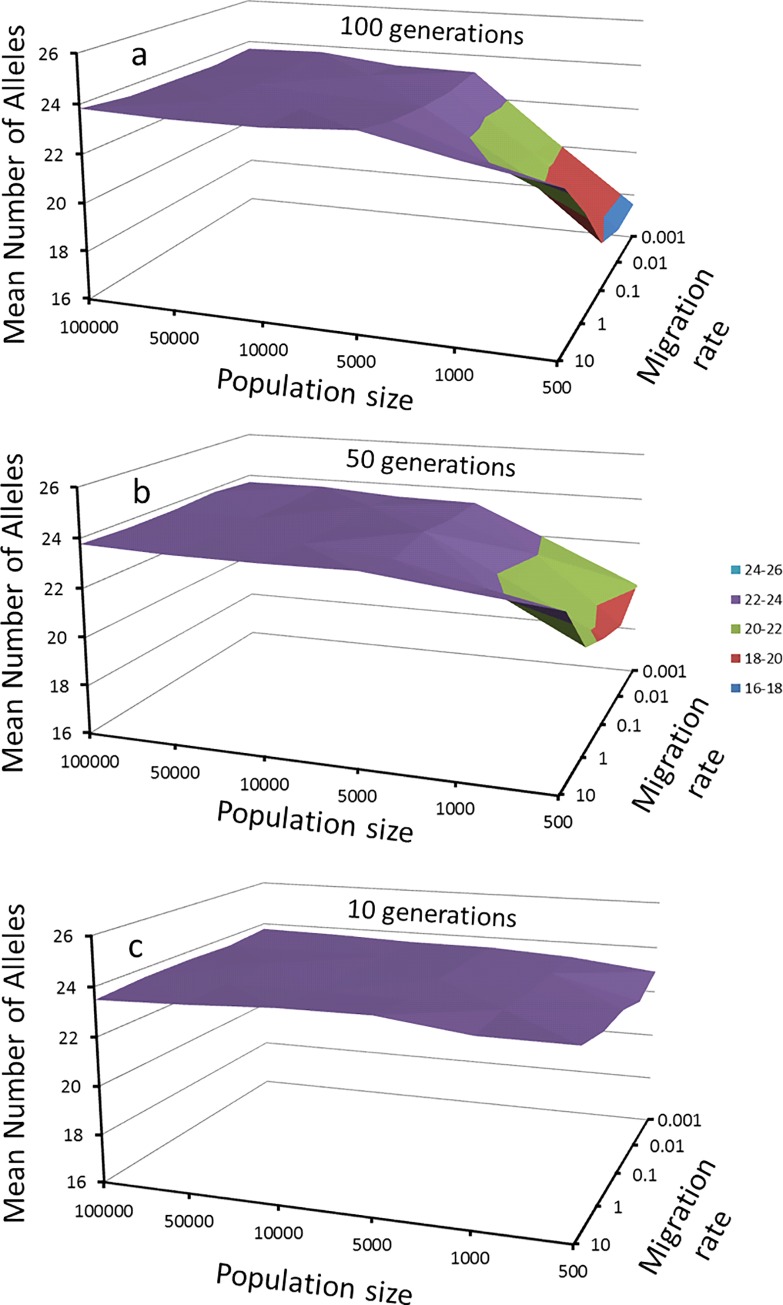
**Reduction in the number of alleles per locus estimated for the simulation of six populations as a function of the population size and migration rate, data after a) 100, b) 50 and c) 10 generations.**

This simulation allows for the observation of different scenarios at different times (see Figs 3 and 4). First, sites containing 100,000 individuals for 100 generations showed no significant differences in the F_ST_ values (Fig 3) and did not reveal a decreasing number of alleles even when the rate of migration was reduced from 10% (observed value for a mutation rate of 1 x 10^−2^) to a rate 1000 times lower. Second, sites with small population sizes showed differences in allele frequencies and lost part of their allelic diversity. After the 10^th^ generation, the sites containing 1,000 individuals and 10% migrants had significantly different F_ST_ values. The same was observed for populations composed of 5,000 individuals and a 0.1% migration rate. Finally, in the worst simulated case (500 individuals and migration rate of 0.001%), a significant value of F_ST_ (F_ST_ = 0.0128, P < 0.05) was observed. This difference was primarily due to changes in allele frequencies, since only one allele per locus was lost during this time. The decrease in the number of alleles appears to be gradual; at the 50th generation the simulation predicted a loss of 4 alleles per locus (16% of the total alleles) and 7 alleles (29%) at the 100^th^ generation (Fig 4).

## Discussion

The genetic analysis performed in *Metacarcinus edwardsii* along 700 km of coastline showed a population genetic structure in accordance with the duration of planktonic larvae. Genetic analyses suggest there is no genetic structure at temporal and spatial levels, with a historical mean of 10% of the progeny migrating every year among sampling sites.

### Temporal and spatial genetic patterns in *M*. *edwardsii*

The traditional *F*_ST_ and the unbiased *G*”_ST_ showed no evidence for spatial and temporal structure in *M*. *edwardsii*; these results were consistent with the analyses performed in both STRUCTURE and FLOCK software. Furthermore, the analysis showed no evidence of isolation by distance. The spatial genetic homogeneity described here for *M*. *edwardsii* is consistent with the long planktonic larval period. Other species with long planktonic development have shown a lack of spatial structure, for example invertebrates in the coast of South Africa [63] and the crustaceans *Chionoecetes opilio* [64] and *Cancer pagurus* [26]. This lack of structure is related to how far larvae can disperse in the early phases of development. Shanks et al. (2003) [65] reported the dispersal distances of different crustaceans, in particular, *Carcinus maenas*, a brachyuran with a larval period of 80 days, can disperse from 63 to 173km depending on the zone studied. Similarly, Kinlan and Gaines (2003) [66] reported that invertebrates with feeding larvae show an average dispersal distance of 100 km.

Due to the long planktonic period, high mortality during this stage of development could be expected [67], which along with the variation of oceanographic conditions might have caused changes in allele frequencies between cohorts. Genetic homogeneity among consecutive cohorts observed in *M*. *edwardsii* is an unexpected result considering that Los Molinos shows seasonal coastal upwelling events [32], and even had a tsunami which led to changes in coastal topography in February, 2010 [68]. While this homogeneity is an unusual pattern among marine invertebrates, the crab *Carcinus maenas* [69] and the bivalve *Panopea generosa* [70] have also shown temporal genetic stability in a similar context of complexity.

### Factors explaining the pattern observed in *M*. *edwardsii*

The biology of *M*. *edwardsii* could explain the observed pattern of genetic structure.

1. Reproductive variance. Genetic homogeneity over time has been associated with species that have low reproductive variance among individuals that compose each population [69]. As was pointed out by Broquet et al. (2013) [71], high reproductive variance is the main factor explaining ephemeral genetic differentiation at small scale (chaotic genetic patchiness) observed in benthic invertebrates. Thus, this low reproductive variance can be explained by characteristics exhibited in the brachyuran crab reproductive system, with capacity to store spermatophores for several months [40]. Therefore, if females do not find mates in one season, sperm transferred during the previous season and stored in seminal receptacles could be used for fertilization. In this condition, all females are able to produce viable broods every year.

2. High larval dispersal. Species with high potential for dispersion given the duration of their pelagic larvae, usually have high levels of connectivity [72]. According to Haye et al. (2014) [31], the time spent by larvae in the water column, as a proxy of the dispersion potential, is the factor that best explains the genetic structure present in marine invertebrates in the transition area (30–33° S) in Chile. *M*. *edwardsii*, with its long larval period, is a good example of a species showing a lack of genetic structure at a wide spatial scale.

3. Complete larval mixing during the planktonic period. It has been widely assumed that during the planktonic period, larvae from different locations and/or parents are completely free to mix (e.g. [73]). However, there is evidence showing that discrete patches of larvae could be maintained in the water column on a scale of days to weeks [74,75], allowing the settlement of related individuals in the same place [76,19,77]. The factors that promote or reduce larval mix are related to oceanographic conditions and the behavior of the females and/or larvae, and not to the potential these species have to disperse [19, 20]. In this context, incomplete larval mix has been pointed to as a secondary factor affecting stochastic changes in allele frequencies [71]. The similar allele frequencies observed each year in *M*. *edwardsii* and the lack of evidence of relatedness within each megalopa cohort suggested that larvae mix completely during the planktonic period before benthic settlement. Another study performed in the brachyuran *Carcinus maenas* also showed genetic stability among cohorts [69].

4. Reproductive output. Species with greater reproductive output show more broad-scale connectivity among populations than species with low reproductive output [78]. Brachyuran species have the highest reproductive output among marine invertebrates. Hines (1991) [79] estimated 18,200 to 2,208,000 eggs per brood in nine *Cancer* crab species on the east coast of the USA. Interestingly, females of the brachyuran *Cancer pagurus* produced between 605,600 to 2,310,000 eggs per brood [79], and had no population structure in the Norwegian Sea [26]. Our measurements, performed on *M*. *edwardsii*, showed values of 500,000 to 1,000,000 eggs per brood.

### Effective population size

Traditionally, estimates of Ne were based on samples of middle age; while samples of one cohort provide information about the *Nb* (model based on semelparous species) [80]. In this case, Ne per generation could be larger than Nb by a factor similar to the generation length (*Ne*≈*G*×*Nb*) [81]. For iteroparous species, Waples et al. (2014) [82] suggested that samples from each cohort provide information to estimate the Nb and also could be used to the estimate *Ne*; however, this requires taking into account a correction that considers some life history traits. In our study, the Nb and Ne had similar values, confirming the analysis of Waples et al. (2014) [82]. Similar results also have been described for the frog *Bufo calamita* [83], the sturgeon *Acipenser fulvescens* [84] and the salamanders *Ambystoma opacum* and *A*. *talpoideum* [85].

### Genetic basis for fisheries management and resource conservation

*M*. *edwardsii* is the most important artisanal crab fishery in Chile; which makes it important to understand the current state of the species in order to understand the effect of the fishery on its future. This study was conducted in the most intense crab fishing area in the country [35] (Concepción, Los Molinos and Calbuco are low fishery intensity localities, while Ancud, Dalcahue and Quellón are high fishery intensity localities), so the conclusions made here are valid for future management plans. Our results indicate that the larvae produced in unexploited areas are naturally connected with exploited areas, aiding to avoid the reduction of stocks and irreversible loss of genetic diversity. Considering that crab fishery increases every year due to the pressure imposed by the international market and reallocation of fishery effort after the depletion of other resources (i.e. finfishes, Chilean abalone), crab landings in the southern Chile have increased in the last decade and stock seems be resilient to increment of effort. High larval subsidies of individuals from unexploited areas could be helping maintain stock. Considering our data and the simulation, the crab fishery should remain an artisanal-only activity [86] and control should be reinforced in effective management measures in order to avoid drastic changes in genetic fluxes.

## Supporting Information

S1 Table Summary of genetic values by sampling site. (DOCX)

S2 Table Summary of genetic values for each megalopa cohort. (DOCX)

## References

[1] Thorson G. Reproductive and larval ecology of marine bottom invertebrates. Biol Rev. 1950; 25:1–45. 24537188 10.1111/j.1469-185x.1950.tb00585.x

[2] Pineda J, Reyns NB, Starczak VR. Complexity and simplification in understanding recruitment in benthic populations. Pop Ecol. 2009; 51:17–32.

[3] Cowen RK, Gawarkiewicz G, Pineda J, Thorrold SR, Werner FE. Population connectivity in marine systems; an overview. Oceanography. 2007; 20:14–21.

[4] Morgan SG, Fisher JL. Larval behavior regulates nearshore retention and offshore migration in an upwelling shadow and along the open coast. Mar Ecol Progr Ser. 2010; 404:109–126.

[5] Yannicelli B, Castro L, Parada C, Schneider W, Colas F, Donoso D. Distribution of *Pleuroncodes monodon* larvae over the continental shelf of south-central Chile: Field and modeling evidence for partial local retention and transport. Prog Oceanogr. 2012; 92:206–227.

[6] Bartilotti C, dos Santos A, Castro M, Peliz A, Santos AMP. Decapod larval retention within distributional bands in a coastal upwelling ecosystem. Mar Ecol Progr Ser. 2014; 507:233–247.

[7] Colson I, Hughes RN. Rapid recovery of genetic diversity of dogwhelk (*Nucella lapillus* L.) populations after local extinction and recolonization contradicts predictions from life‐history characteristics. Mol Ecol. 2004; 13:2223–2233. 10.1111/j.1365-294X.2004.02245.x 15245396

[8] Waters JM, Roy MS. Phylogeography of a high‐dispersal New Zealand sea‐star: does upwelling block gene‐flow? Mol Ecol. 2004; 13:2797–2806. 10.1111/j.1365-294X.2004.02282.x 15315690

[9] Kyle CJ, Boulding EG. Comparative population genetic structure of marine gastropods (*Littorina* spp.) with and without pelagic larval dispersal. Mar Biol. 2000; 137:835–845.

[10] Shanks AL, Shearman RK. Paradigm lost? Cross-shelf distributions of intertidal invertebrate larvae are unaffected by upwelling or downwelling. Mar Ecol Progr Ser. 2009; 385:189–204.

[11] Queiroga H, Cruz T, dos Santos A, Dubert J, González-Gordillo JI, Paula J et al Oceanographic and behavioural processes affecting invertebrate larval dispersal and supply in the western Iberia upwelling ecosystem. Progr Oceanogr. 2007; 74:174–191.

[12] Pardo LM, Cardyn CS, Garcés-Vargas J. Spatial variation in the environmental control of crab larval settlement in a micro-tidal austral estuary. Helgoland Mar Res. 2012a; 66:253–263.

[13] Cowen RK, Sponaugle S. Larval dispersal and marine population connectivity. Annu Rev Mar Sci. 2009; 1:443–466. 10.1146/annurev.marine.010908.163757 21141044

[14] Robainas Barcia A, Espinosa López G, Hernández D, García-Machado E. Temporal variation of the population structure and genetic diversity of *Farfantepenaeus notialis* assessed by allozyme loci. Mol Ecol. 2005; 14:2933–2942. 10.1111/j.1365-294X.2005.02613.x 16101764

[15] Barshis DJ, Sotka EE, Kelly RP, Sivasundar A, Menge BA, Barth JA et al Coastal upwelling is linked to temporal genetic variability in the acorn barnacle *Balanus glandula*. Mar Ecol Progr Ser. 2011; 439:139–150.

[16] Johnson MS, Black R. Chaotic genetic patchiness in an intertidal limpet, *Siphonaria* sp. Mar Biol. 1982; 70:157–164.

[17] Johnson MS, Black R. Pattern beneath the chaos: the effect of recruitment on genetic patchiness in an intertidal limpet. Evolution. 1984; 38:1371–1383. 28563786 10.1111/j.1558-5646.1984.tb05658.x

[18] Lee HJE, Boulding EG. Spatial and temporal population genetic structure of four northeastern Pacific littorinid gastropods: the effect of mode of larval development on variation at one mitochondrial and two nuclear DNA markers. Mol Ecol. 2009; 18:2165–2184. 10.1111/j.1365-294X.2009.04169.x 19344352

[19] Veliz D, Duchesne P, Bourget E, Bernatchez L. Genetic evidence for kin aggregation in the intertidal acorn barnacle (*Semibalanus balanoides*). Mol Ecol. 2006; 15:4193–4202. 10.1111/j.1365-294X.2006.03078.x 17054512

[20] Iacchei M, Ben-Horin T, Selkoe KA, Bird KE, Garcia-Rodriguez FJ, Toonen RJ. Combined analyses of kinship and F_ST_ suggest potential drivers of chaotic genetic patchiness in high gene-flow populations. Mol Ecol. 2013; 22:3476–3494. 10.1111/mec.12341 23802550 PMC3749441

[21] Therkildsen NO, Nielsen EE, Swain DP, Pedersen JS. Large effective population size and temporal genetic stability in Atlantic cod (*Gadus morhua*) in the southern Gulf of St. Lawrence. Can J Fish Aquat Sci. 2010; 67:1585–1595.

[22] Frankham R, Ballou JD, Briscoe DA. Introduction to Conservation Genetics. Cambridge University Press, Cambridge, UK 2002.

[23] Smith PJ, Francis R, McVeagh M. Loss of genetic diversity due to fishing pressure. Fish Res. 1991; 10:309–316.

[24] Hauser L, Adcock GJ, Smith PJ, Ramirez JHB, Carvalho GR. Loss of microsatellite diversity and low effective population size in an overexploited population of New Zealand snapper (*Pagrus auratus*). Proc Natl Acad Sci USA. 2002; 99:11742–11747. 10.1073/pnas.172242899 12185245 PMC129339

[25] Poulsen NA, Nielsen EE, Schierup MH, Loeschcke V, Grønkjaer P. Long-term stability and effective population size in North Sea and Baltic Sea cod (*Gadus morhua*). Mol Ecol. 2006; 15:321–331. 10.1111/j.1365-294X.2005.02777.x 16448403

[26] Ungfors A, McKeown NJ, Shaw PW, Andre C. Lack of spatial genetic variation in the edible crab (*Cancer pagurus*) in the Kattegat–Skagerrak area. ICES J Mar Sci. 2009; 66:462–469.

[27] Kelly RP, Palumbi SR. Genetic structure among 50 species of the Northeastern Pacific rocky intertidal community. PLoS One. 2010; 5:e8594 10.1371/journal.pone.0008594 20062807 PMC2799524

[28] Thiel M, Macaya E, Acuña E, Arntz W, Bastias H, Brokordt K et al The Humboldt Current System of northern and central Chile: oceanographic processes, ecological interactions and socioeconomic feedback. Oceanogr Mar Biol. 2007; 45:195–345.

[29] Camus PA. Biogeografía marina de Chile continental. Rev Chil Hist Nat. 2001; 74:587–617. Spanish.

[30] Laughlin KM, Ewers C, Wares JP. Mitochondrial lineages in *Notochthamalus scabrosus* as indicators of coastal recruitment and interactions. Ecol Evol. 2012; 2:1584–1591. 10.1002/ece3.283 22957164 PMC3434930

[31] Haye PA, Segovia NI, Muñoz-Herrera NC, Gálvez FE, Martínez A, Meynard A et al Phylogeographic structure in benthic marine invertebrates of the southeast Pacific coast of Chile with differing dispersal potential. PloS One. 2014; 9:e88613 10.1371/journal.pone.0088613 24586356 PMC3929388

[32] Cárdenas L, Castilla JC, Viard F. A phylogeographical analysis across three biogeographical provinces of the south‐eastern Pacific: the case of the marine gastropod *Concholepas concholepas*. J Biogeogr. 2009; 36:969–981.

[33] Vinuesa JH, Lovrich GA, Tapella F. New localities for Crustacea Decapoda in the Magellan Region, southern South America. Sci Mar. 1999; 63:321–323.

[34] SERNAPESCA. Anuarios Estadísticas de Pesca. Servicio Nacional de Pesca y Acuicultura de Chile, Ministerio de Economía, Fomento y Turismo, Gobierno de Chile. Anuario 2011—Series 2001–2011. Spanish.

[35] Pardo LM, Rosas Y, Fuentes JP, Riveros MP, Chaparro OR. Fishery Induces Sperm Depletion and Reduction in Male Reproductive Potential for Crab Species under Male-Biased Harvest Strategy. PLoS One. 2015; 10:e0115525 10.1371/journal.pone.0115525 25768728 PMC4359117

[36] Quintana R. Larval development of the edible crab, *Cancer edwardsi* Bell, 1835 under laboratory conditions (Decapoda: Brachyura). Rep USA Mar Biol Inst Kochi Univ. 1983; 5: 1–19.

[37] Pardo LM, Mora-Vásquez P, Garcés-Vargas J. Asentamiento diario de megalopas de jaibas del género *Cancer* en un estuario micromareal. Lat Am J Aquat Res. 2012b; 40:142–152. Spanish.

[38] Jara F, Céspedes R. An experimental evaluation of habitat enhancement on homogeneous marine bottoms in southern Chile. Bull Mar Sci. 1994; 55:295–307.

[39] Pardo LM, Fuentes JP, Olguin A, Orensanz JM. Reproductive maturity in the edible Chilean crab *Cancer edwardsii*, methodological and management considerations. J Mar Biol Assoc UK. 2009a; 89:1627–1634.

[40] Pardo LM, Riveros M, Fuentes JP, Rojas-Hernandez N, Veliz D. An effective sperm competition avoidance strategy in crabs drives genetic monogamy despite evidence of polyandry. Behav Ecol Sociobiol. 2016; 70:73–81.

[41] Pardo LM, Ampuero D, Véliz D. Using morphological and molecular tools to identify megalopae larvae collected in the field: the case of sympatric Cancer crabs. J Mar Biol Assoc UK. 2009b; 89:481–490.

[42] Pardo LM, Cardyn CS, Mora P, Wahle RA. A new passive collector to assess settlement rates, substrate selection and predation pressure in decapod crustacean larvae. J Exp Mar Biol Ecol. 2010; 393:100–105.

[43] Aljanabi SM, Martinez I. Universal and rapid salt-extraction of high quality genomic DNA for PCR-based techniques. Nucleic Acid Res. 1997; 25:4692–4693. 9358185 10.1093/nar/25.22.4692 PMC147078

[44] Rojas-Hernández N, Véliz D, Pardo LM. Use of novel microsatellite markers for population and paternity analysis in the commercially important crab *Metacarcinus edwardsii* (Brachyura: Cancridae). Mar Biol Res. 2014; 10:839–844.

[45] Belkhir K, Borsa P, Chikhi L, Raufaste N, Bonhomme F. GENETIX 4.05, Logiciel sous Windows pour la Genetique des Populations. Laboratoire Genome, populations, interactions, CNRS UMR 5000, Universite de Montpellier II, Montpellier, France 1996. French.

[46] Goudet J. FSTAT (version 1.2)–a computer program to calculate F-statistics. J Hered. 1995; 86:485–486.

[47] van Oosterhout C, Hutchinson WF, Wills DPM, Shipley P. Microchecker: software for identifying and correcting genotyping errors in microsatellite data. Mol Ecol Notes. 2004; 4:535–538.

[48] Queller DC, Goodnight KF. Estimating relatedness using genetic markers. Evolution. 1989; 43:258–275. 28568555 10.1111/j.1558-5646.1989.tb04226.x

[49] Belkhir K, Castric V, Bonhomme F. IDENTIX, a software to test for relatedness in a population using permutation methods. Mol Ecol Notes. 2001; 2:611–614.

[50] Meirmans P, Hedricks PW. Assessing population structure: FST and related measures. Mol Ecol Resur. 2011; 11:5–18. 10.1111/j.1755-0998.2010.02927.x 21429096

[51] Peakall R, Smouse PE. GenAlEx 6.5: genetic analysis in Excel. Population genetic software for teaching and research-an update. Bioinformatics. 2012; 28:2537–2539. 10.1093/bioinformatics/bts460 22820204 PMC3463245

[52] Pritchard JK, Stephens M, Donnelly P. Inference of population structure using multilocus genotype data. Genetics. 2000; 115:945–959. 10.1093/genetics/155.2.945 PMC1461096 10835412

[53] Duchesne P, Turgeon J. FLOCK attractors provide reliable solutions to the “number of populations” problem. J Hered. 2012; 103:734–743. 10.1093/jhered/ess038 22615162

[54] Earl DA, von Holdt BM. STRUCTURE HARVESTER: a website and program for visualizing STRUCTURE output and implementing the Evanno method. Conserv Genet Res. 2012; 4:359–361.

[55] Paetkau D, Calvert W, Sterling I, Strobeck C. Microsatellite analysis of population-structure in Canadian polar bears. Mol Ecol. 1995; 4:347–354. 7663752 10.1111/j.1365-294x.1995.tb00227.x

[56] Beerli P, Felsenstein J. Maximum likelihood estimation of a migration matrix and effective population sizes in n subpopulations by using a coalescent approach. Proc Natl Acad Sci USA. 2001; 98:4563–4568. 10.1073/pnas.081068098 11287657 PMC31874

[57] Tallmon DA, Koyuk A, Luikart G, Beaumont MA. Onesamp: a program to estimate effective population size using approximate Bayesian computation. Mol Ecol Resur. 2008; 8:299–301. 10.1111/j.1471-8286.2007.01997.x 21585773

[58] Do C, Waples RS, Peel D, Macbeth GM, Tillett BJ, Ovenden JR. NeEstimator V2: re-implementation of software for the estimation of contemporary effective population size (Ne) from genetic data. Mol Ecol Resour. 2014; 14:209–214. 10.1111/1755-0998.12157 23992227

[59] Cornuet JM, Luikart G. Description and power analysis of two tests for detecting recent population bottlenecks from allele frequency data. Genetics. 1996; 144:2001–2014. 8978083 10.1093/genetics/144.4.2001 PMC1207747

[60] Waples RS, Do C. Linkage disequilibrium estimates of contemporary Ne using highly variable genetic markers: a largely untapped resource for applied conservation and evolution. Evol Appl. 2010; 3:244–262. 10.1111/j.1752-4571.2009.00104.x 25567922 PMC3352464

[61] Roughgarden J, Gaines S, Possingham H. Recruitment dynamics in complex life cycles. Science. 1988; 241:1460–1466. 11538249 10.1126/science.11538249

[62] Balloux F. EASYPOP (version 1.7) A computer program for the simulation of population genetics. J Hered. 2001; 92:301–302. 11447253 10.1093/jhered/92.3.301

[63] Teske PR, Papadopoulos I, Zardi GI, McQuaid CD, Edkins MT, Griffiths CL et al Implications of life history for genetic structure and migration rates of southern African coastal invertebrates: planktonic, abbreviated and direct development. Mar Biol. 2007; 152:697–711.

[64] Puebla O, Sévigny JM, Sainte-Marie B, Brêthes JC, Burmeister A, Dawe EG et al Population genetic structure of the snow crab (*Chionoecetes opilio*) at the Northwest Atlantic scale. Can J Fish Aquat Sci. 2008; 65:425–436.

[65] Shanks AL, Grantham BA, Carr MH. Propagule dispersal distance and the size and spacing of marine reserves. Ecological applications. 2003; 159–169.

[66] Kinlan BP, Gaines SD. Propagule dispersal in marine and terrestrial environments: a community perspective. Ecology. 2003; 2007–2020.

[67] Morgan SG. Life and death in the plankton: larval mortality and adaptation In: McEdward L (ed) Ecology of marine invertebrate larvae. CRC Press, Boca Raton, Florida 1995; p 279–321.

[68] Farias M, Vargas G, Tassara A, Carretier S, Baize S, Melnick D et al Land-level changes produced by the Mw 8.8 2010 Chilean earthquake. Science. 2011; 329:916. 10.1126/science.1192094 20671154

[69] Domingues CP, Creer S, Taylor MI, Queiroga H, Carvalho GR. Temporal genetic homogeneity among shore crab (*Carcinus maenas*) larval events supplied to an estuarine system on the Portuguese northwest coast. Heredity. 2011; 106:832–840. 10.1038/hdy.2010.126 20959862 PMC3186222

[70] Vadopalas B, Leclair LL, Bentzen P. Temporal genetic similarity among year-classes of the Pacific geoduck clam (*Panopea generosa* Gould 1850): a species exhibiting spatial genetic patchiness. J Shellfish Res. 2012; 31:697–709.

[71] Broquet T, Viard F, Yearsley JM. Genetic drift and collective dispersal can result in chaotic genetic patchiness. Evolution. 2013; 67:1660–1675. 10.1111/j.1558-5646.2012.01826.x 23730760

[72] Siegel DA, Kinlan BP, Gaylord B, Gaines SD. Langrangian descriptions of marine larval dispersion. Mar Ecol Progr Ser. 2003; 260: 83–96.

[73] Le Fevre J, Bourget E. Hydrodynamics and behavior: transport process in marine invertebrate larvae. TREE. 1992; 7:288–289. 10.1016/0169-5347(92)90223-X 21236035

[74] Kordos LM, Burton RS. Genetic differentiation of Texas Gulf coast populations of the blue crab *Callinectes sapidus*. Mar Biol. 1993; 117:227–233.

[75] Natunewicz CC, Epifanio CE. Spatial and temporal scales of patches of crab larvae in coastal waters. Mar Ecol Progr Ser. 2001; 212:217–222.

[76] Selkoe KA, Gaines SD, Caselle JE, Warner RR. Current shifts and kin aggregation explain genetic patchiness in fish recruits. Ecology. 2006; 87:3082–3094. 17249233 10.1890/0012-9658(2006)87[3082:csakae]2.0.co;2

[77] St-Onge P, Tremblay R, Sévigny JM. Tracking larvae with molecular markers reveals high relatedness and early seasonal recruitment success in a partially spawning marine bivalve. Oecologia. 2015; 178: 733–746. 10.1007/s00442-015-3245-2 25715923

[78] Treml EA, Roberts JJ, Chao YI, Halpin PN, Possingham HP, Riginos C. Reproductive output and duration of the pelagic larval stage determine seascape-wide connectivity of marine populations. Integr Comp Biol. 2012; 52:525–537. 10.1093/icb/ics101 22821585

[79] Hines AH. Fecundity and reproductive output in nine species of Cancer crabs (Crustacea: Brachyura: Cancridae). Can J Fish Aquat Sci. 1991; 48:267–275.

[80] Waples RS, Teel DJ. Conservation genetics of Pacific salmon I. Temporal changes in allele frequency. Conserv Biol. 1990; 4:144–156.

[81] Nunney L. The effective size of annual plant populations: the interaction of a seed bank with fluctuating population size in maintaining genetic variation. Am Nat. 2002; 160:195–204. 10.1086/341017 18707486

[82] Waples RS, Antao T, Luikart G. Effects of overlapping generations on linkage disequilibrium estimates of effective population size. Genetics. 2014; 197:769–780. 10.1534/genetics.114.164822 24717176 PMC4063931

[83] Beebee TJC. A comparison of single‐sample effective size estimators using empirical toad (*Bufo calamita*) population data: genetic compensation and population size‐genetic diversity correlations. Mol Ecol. 2009; 18:4790–4797. 10.1111/j.1365-294X.2009.04398.x 19863715

[84] Duong TY, Scribner KT, Forsythe PS, Crossman JA, Baker EA. Interannual variation in effective number of breeders and estimation of effective population size in long‐lived iteroparous lake sturgeon (*Acipenser fulvescens*). Mol Ecol. 2013; 22:1282–1294. 10.1111/mec.12167 23293919

[85] Nunziata SO, Scott DE, Lance SL. Temporal genetic and demographic monitoring of pond-breeding amphibians in three contrasting population systems. Conserv Genet. 2015; 16:1–10.

[86] Pauly D, Christensen V, Guénette S, Pitcher TJ, Sumaila UR, Walters CJ et al Towards sustainability in world fisheries. Nature. 2002; 418:689–695. 10.1038/nature01017 12167876

