# Supplementary material for: Highly Connected Populations and Temporal Stability in Allelic Frequencies of a Harvested Crab from the Southern Pacific Coast
Source: PLoS One. 2016 Nov 4;11(11):e0166029. doi: 10.1371/journal.pone.0166029 (PMC5096711; doi:10.1371/journal.pone.0166029)
Supplement: S1 Table — (DOCX) [file pone.0166029.s001.docx]

**S1 Table.** **Summary of genetic values per sampling site.** Sample size (N), number of alleles (Na), allelic richness (Ar), expected heterozygosity (H_E_), observed heterozygosity (H_O_), F_IS_ according to Weir and Cockerham (1984). Values in bold indicate samples which deviate significantly from HWE. The last lines show the relatedness index (r_xy_) estimated per sampling site and the p-value.

|  | Sampling sites | | | | | |
| --- | --- | --- | --- | --- | --- | --- |
|  | LM | DA | AN | CA | QU | CO |
| *Cedw15* |  |  |  |  |  |  |
| N | 43 | 48 | 59 | 29 | 73 | 26 |
| Na | 8 | 7 | 7 | 7 | 7 | 5 |
| Ar | 7.241 | 6.534 | 6.441 | 6.351 | 6.189 | 4.98 |
| H_E_ | 0.682 | 0.682 | 0.599 | 0.617 | 0.589 | 0.558 |
| H_O_ | 0.744 | 0.688 | 0.576 | 0.586 | 0.616 | 0.615 |
| Fis | -0.079 | 0.003 | 0.047 | 0.067 | -0.039 | -0.084 |
| *Cedcrab1* |  |  |  |  |  |  |
| N | 43 | 47 | 61 | 31 | 76 | 25 |
| Na | 17 | 20 | 20 | 17 | 19 | 14 |
| Ar | 14.731 | 16.058 | 14.747 | 14.923 | 14.35 | 13.27 |
| H_E_ | 0.917 | 0.925 | 0.916 | 0.915 | 0.915 | 0.893 |
| H_O_ | 0.861 | 0.872 | 0.853 | 0.903 | 0.842 | 0.840 |
| Fis | 0.073 | 0.068 | 0.077* | 0.029 | 0.086* | 0.079 |
| *Cedcrab4* |  |  |  |  |  |  |
| N | 44 | 40 | 61 | 32 | 76 | 26 |
| Na | 23 | 17 | 24 | 20 | 26 | 16 |
| Ar | 15.703 | 14.08 | 17.183 | 16.687 | 17.043 | 15.077 |
| H_E_ | 0.911 | 0.910 | 0.925 | 0.919 | 0.930 | 0.919 |
| H_O_ | 0.886 | 0.800 | 0.934 | 0.938 | 0.921 | 1.000 |
| Fis | 0.038 | 0.133* | -0.002 | -0.005 | 0.016 | -0.069 |
| *Cedw16* |  |  |  |  |  |  |
| N | 31 | 38 | 60 | 32 | 73 | 20 |
| Na | 31 | 34 | 36 | 25 | 35 | 24 |
| Ar | 24.603 | 25.133 | 23.336 | 20.329 | 21.84 | 24 |
| H_E_ | 0.956 | 0.961 | 0.958 | 0.936 | 0.952 | 0.950 |
| H_O_ | 0.871 | 0.947 | 0.950 | 0.938 | 0.959 | 0.850 |
| Fis | 0.105* | 0.027 | 0.016 | 0.014 | -0.001 | 0.131* |
| *Cedw4* |  |  |  |  |  |  |
| N | 43 | 40 | 51 | 23 | 57 | 24 |
| Na | 40 | 37 | 38 | 27 | 41 | 27 |
| Ar | 26.463 | 25.816 | 25.205 | 25.041 | 25.763 | 24.338 |
| H_E_ | 0.965 | 0.963 | 0.964 | 0.953 | 0.966 | 0.950 |
| H_O_ | 0.930 | 0.900 | 0.922 | 0.870 | 0.895 | 0.958 |
| Fis | 0.048 | 0.078* | 0.053* | 0.109* | 0.082* | 0.012 |
| *Cedcrab3* |  |  |  |  |  |  |
| N | 43 | 46 | 56 | 28 | 54 | 26 |
| Na | 36 | 30 | 41 | 27 | 34 | 24 |
| Ar | 23.61 | 20.731 | 24.528 | 22.684 | 22.767 | 20.737 |
| H_E_ | 0.948 | 0.940 | 0.961 | 0.946 | 0.957 | 0.933 |
| H_O_ | 0.907 | 0.804 | 0.946 | 0.929 | 0.907 | 0.962 |
| Fis | 0.055 | 0.155* | 0.024 | 0.037 | 0.061* | -0.011 |
| *Cedw5* |  |  |  |  |  |  |
| N | 32 | 36 | 56 | 28 | 65 | 24 |
| Na | 26 | 28 | 33 | 27 | 34 | 27 |
| Ar | 21.11 | 21.634 | 21.566 | 23.026 | 22.87 | 24.134 |
| H_E_ | 0.943 | 0.947 | 0.952 | 0.948 | 0.960 | 0.940 |
| H_O_ | 0.844 | 0.806 | 0.893 | 0.893 | 0.939 | 0.875 |
| Fis | 0.121* | 0.163* | 0.071* | 0.077 | 0.030 | 0.090 |
| *Cedw12* |  |  |  |  |  |  |
| N | 45 | 48 | 55 | 31 | 73 | 26 |
| Na | 16 | 21 | 20 | 18 | 21 | 15 |
| Ar | 13.817 | 15.052 | 15.719 | 15.382 | 15.263 | 13.798 |
| H_E_ | 0.917 | 0.915 | 0.921 | 0.906 | 0.911 | 0.861 |
| H_O_ | 0.956 | 0.958 | 0.891 | 0.903 | 0.959 | 0.846 |
| Fis | -0.031 | -0.036 | 0.042 | 0.019 | -0.046 | 0.037 |
| r_xy_ | -0.023 | -0.023 | -0.018 | -0.036 | -0.014 | -0.042 |
| P | 0.171 | 0.380 | 0.551 | 0.706 | 0.316 | 0.550 |
