# Supplementary material for: Highly Connected Populations and Temporal Stability in Allelic Frequencies of a Harvested Crab from the Southern Pacific Coast
Source: PLoS One. 2016 Nov 4;11(11):e0166029. doi: 10.1371/journal.pone.0166029 (PMC5096711; doi:10.1371/journal.pone.0166029)
Supplement: S2 Table — (DOCX) [file pone.0166029.s002.docx]

**S2 Table.** **Summary of genetic values per cohort of megalopa.** Sample size (N), number of alleles (Na), allelic richness (Ar), expected heterozygosity (H_E_), observed heterozygosity (H_O_), F_IS_ according to Weir and Cockerham (1984). Values in bold indicate samples which deviate significantly from HWE. The last lines show the relatedness index (r_xy_) estimated per sampling site and the p-value.

|  | sampling year | | | |
| --- | --- | --- | --- | --- |
|  | 2009 | 2010 | 2011 | 2012 |
| *Cedw15* |  |  |  |  |
| N | 34 | 51 | 37 | 34 |
| Na | 7 | 7 | 6 | 7 |
| Ar | 5.485 | 4.865 | 4.394 | 4.927 |
| H_E_ | 0.714 | 0.627 | 0.634 | 0.668 |
| H_O_ | 0.794 | 0.686 | 0.595 | 0.706 |
| Fis | -0.098 | -0.085 | 0.076 | -0.042 |
| *Cedcrab1* |  |  |  |  |
| N | 35 | 50 | 37 | 34 |
| Na | 19 | 18 | 20 | 18 |
| Ar | 10.067 | 10.013 | 10.741 | 9.782 |
| H_E_ | 0.916 | 0.920 | 0.929 | 0.902 |
| H_O_ | 0.886 | 0.860 | 0.892 | 0.765 |
| Fis | 0.048 | 0.075 | 0.054 | 0.167* |
| *Cedcrab4* |  |  |  |  |
| N | 34 | 48 | 36 | 33 |
| Na | 15 | 20 | 17 | 18 |
| Ar | 9.236 | 9.536 | 8.897 | 9.723 |
| H_E_ | 0.904 | 0.909 | 0.887 | 0.908 |
| H_O_ | 0.647 | 0.854 | 0.667 | 0.818 |
| Fis | 0.298* | 0.070 | 0.262* | 0.114* |
| *Cedw16* |  |  |  |  |
| N | 14 | 18 | 8 | 11 |
| Na | 18 | 19 | 12 | 15 |
| Ar | 12.386 | 11.636 | 12 | 11.805 |
| H_E_ | 0.931 | 0.928 | 0.898 | 0.901 |
| H_O_ | 0.857 | 0.722 | 0.875 | 0.909 |
| Fis | 0.116 | 0.248* | 0.093 | 0.038 |
| *Cedcrab3* |  |  |  |  |
| N | 25 | 36 | 20 | 17 |
| Na | 29 | 30 | 25 | 18 |
| Ar | 12.841 | 12.302 | 13.243 | 11.91 |
| H_E_ | 0.946 | 0.948 | 0.950 | 0.933 |
| H_O_ | 0.920 | 0.917 | 0.900 | 0.824 |
| Fis | 0.048 | 0.047 | 0.078 | 0.147* |
| *Cedw5* |  |  |  |  |
| N | 26 | 41 | 21 | 26 |
| Na | 22 | 27 | 20 | 21 |
| Ar | 11.995 | 12.05 | 11.998 | 11.415 |
| H_E_ | 0.942 | 0.948 | 0.938 | 0.933 |
| H_O_ | 0.846 | 0.927 | 0.857 | 0.885 |
| Fis | 0.121* | 0.035 | 0.110* | 0.072 |
| *Cedw12* |  |  |  |  |
| N | 28 | 43 | 30 | 33 |
| Na | 18 | 17 | 17 | 18 |
| Ar | 9.52 | 9.578 | 10.478 | 9.605 |
| H_E_ | 0.892 | 0.911 | 0.926 | 0.907 |
| H_O_ | 0.929 | 0.930 | 0.900 | 1.000 |
| Fis | -0.023 | -0.009 | 0.045 | -0.088 |
| r_xy_ | -0.036 | -0.028 | -0.033 | -0.035 |
| P | 0.732 | 0.873 | 0.593 | 0.686 |
